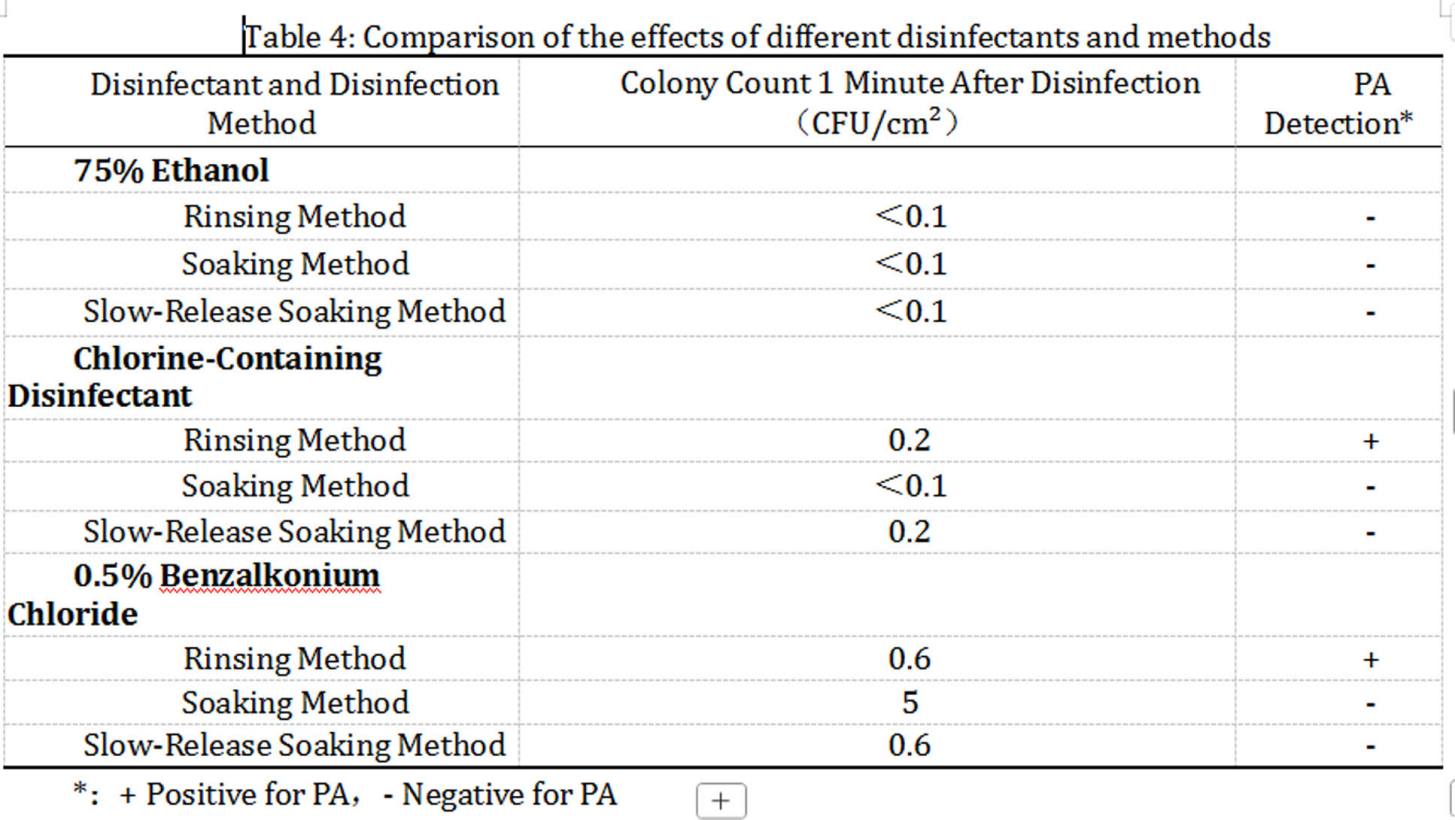# 213 Post-Admission Dwell Time of Non-Sterile vs Sterile Emergency Department–Placed Central Lines

**DOI:** 10.1017/ash.2026.10597

**Published:** 2026-06-23

**Authors:** Dan Liao

**Affiliations:** 1 China

## Abstract

**Objective:** To address the recurrent contamination of handwashing sinks with Pseudomonas aeruginosa (PA) in the Pediatric Intensive Care Unit (PICU), evaluate the PA elimination efficacy of different disinfectants and disinfection methods, and optimize disinfection strategies to prevent PA colonization and transmission, thereby reducing the risk of healthcare-associated infections (HAIs)? **Methods:** Nine handwashing sinks in the PICU (August–December 2024) were selected, meeting the criteria of three consecutive positive PA detections, usage frequency ≥20 times/day, and no prior special disinfection interventions (excluding those with recent pipe replacement or structural damage). They were divided into three groups (3 sinks per group). Three disinfectants were prepared: 500 mg/L chlorine-containing disinfectant, 0.5% benzalkonium chloride disinfectant, and 75% ethanol stock solution. Three disinfection methods were applied: rinsing method (disinfectant flushing for 1 minute twice daily at 5 L/min), soaking method (full coverage soaking with disinfectant for 30 minutes every morning followed by flushing), and slow-release soaking method (slow injection of disinfectant into the trap for 30-minute soaking, 10-minute standing, then flushing at 2 L/min). Samples were collected from the overflow outlet, drain, and other sites before disinfection, 1 minute after disinfection, and 24 hours after disinfection. Colony counting was performed using the pour plate method, PA was identified via the VITEK 2 system and mass spectrometry, and statistical analysis was conducted with SPSS 26.0 (α=0.05). **Results:** 75% ethanol showed the best immediate effect (colony count <0.1 CFU/cm² and PA detection rate 0% 1 minute after disinfection, P<0.001) but PA reoccurred at 24 hours. Chlorine-containing disinfectant performed stably (colony count reduced to 0.2 CFU/cm², PA detection rate 0%, P=0.002) with the optimal 24-hour bacteriostatic effect. Benzalkonium chloride had weak efficacy (colony count reduced to 5.0 CFU/cm², PA detection rate 33.3%). The soaking method and slow-release soaking method were significantly more effective in biofilm removal than the rinsing method (e.g., no PA detected with chlorine-containing disinfectant soaking method and <10% recurrence rate at 24 hours, compared to 66.7% PA positivity rate with the rinsing method at 24 hours), and the soaking method was more operable. **Conclusion:** For PA-contaminated handwashing sinks in the PICU, the chlorine-containing disinfectant soaking method has the best comprehensive effect, combining strong bactericidal power, good long-term bacteriostatic effect, and high operability. It is suitable as a routine disinfection scheme to prevent PA colonization in the ward, providing support for HAI prevention and control. Future research can explore more disinfection methods for ward sinks.

**Figure:**
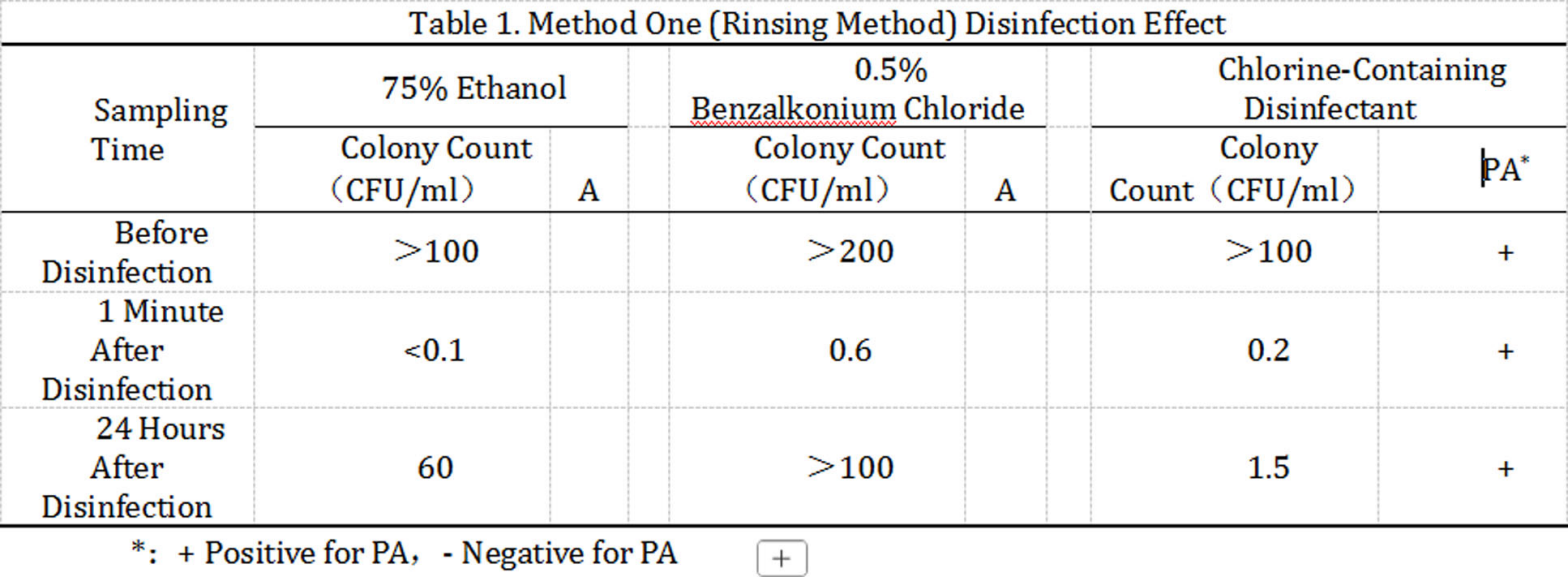

**Figure:**
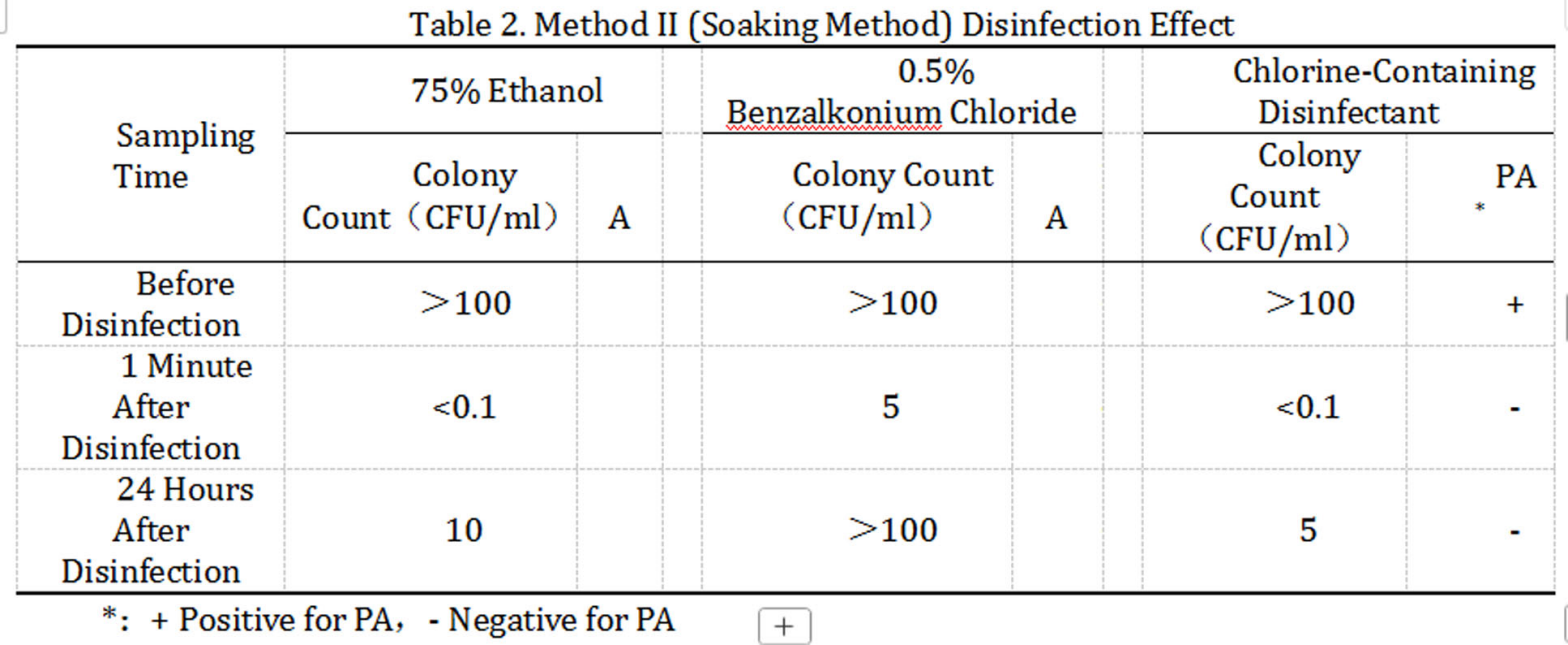

**Figure:**
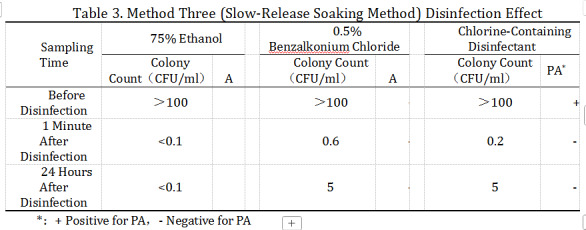

**Figure:**